# Clinical Characteristics and Delays in the Care Pathway of Patients With Lung Cancer Treated at a Public Hospital in Costa Rica, 2015-2020

**DOI:** 10.7759/cureus.116285

**Published:** 2026-09-15

**Authors:** Rebeca Alvarado-Prado, Adolfo Ortiz-Barboza, Alejandro Blanco-Saborío, Julio César Arguello-Méndez, Esteban Zavaleta-Monestel

**Affiliations:** 1 Health Promotion and Epidemiology, Universidad de Costa Rica, San Jose, CRI; 2 Public Health and Bioethics, Universidad de Costa Rica, San Jose, CRI; 3 Radiation Oncology, Caja Costarricense de Seguro Social, San Jose, CRI; 4 Research Department, Hospital Clínica Bíblica, San Jose, CRI

**Keywords:** delayed diagnosis, health inequities, lung cancer, molecular biomarkers, survival, targeted therapy, time to treatment

## Abstract

Introduction: Delays in the lung cancer care pathway and limited access to molecular biomarkers, targeted therapies, and immunotherapy may affect diagnosis, treatment opportunities, and survival. This study described the clinical profile, care pathway delays, biomarker testing, treatment access, and survival of patients with primary lung cancer treated at a public hospital in Costa Rica.

Methods: We conducted a retrospective observational study based on medical record review. Adult patients with a pathological diagnosis of primary lung cancer treated at a public tertiary-level hospital in Costa Rica between 2015 and 2020 were included (n=223). Clinical, sociodemographic, and care process variables were analyzed, including symptoms at the time of consultation, smoking history, diagnostic and therapeutic time intervals, molecular biomarker testing, treatments received, and survival from the first care episode related to suspected lung cancer until death or the end of follow-up.

Results: The most frequent symptoms before diagnosis were cough (n=126; 56.50%), dyspnea (n=110; 49.33%), and weight loss (n=98; 43.95%). The median time between documented suspicion and pathological diagnosis was 34 days (IQR: 21-73; n=213), although extreme values exceeding 1000 days were observed. Molecular biomarker testing was recorded in 106 patients (47.53%), and targeted therapy or immunotherapy was documented in 45 patients (30.00%). Among patients who received targeted therapy or immunotherapy, 29 (64.44%) accessed these treatments through administrative requests to higher institutional authorities outside the standard care protocol. The estimated five-year survival from the first care episode related to suspected lung cancer was 1.9% (95% CI: 0.5-5.1).

Conclusions: This cohort showed frequent diagnostic delays, high variability in care pathway intervals, limited access to molecular biomarkers and innovative therapies, and low five-year survival from the first care episode related to suspected lung cancer. These findings support the need to strengthen suspicion, referral, timely diagnosis, and equitable access to diagnostic technologies and therapeutic alternatives for patients with lung cancer.

## Introduction

The recognition of cancer as a public health problem has generated significant advances in oncological care. However, important inequalities persist across populations and health systems. Addressing these inequities requires specific interventions and local data to guide public policy; in this context, research can contribute to reducing gaps and improving equitable access to oncology services [1].

Lung cancer is one of the leading causes of cancer-related death worldwide. Despite diagnostic and therapeutic advances, many cases are detected at advanced stages, which limits treatment options and reduces survival. Five-year survival generally ranges between 10% and 20%, although it varies according to stage, histological subtype, and access to timely treatment [2].

Although chronic smoking remains the main risk factor for lung cancer, the importance of passive smoking is also recognized [3]. A meta-analysis in the Chinese population estimated that a relevant proportion of lung cancer cases among never-smokers could be attributable to exposure to secondhand tobacco smoke [4].

In addition to smoking, several environmental factors have been associated with lung cancer risk [5]. Therefore, public policy strategies should not be limited to individual lifestyles but should also consider environmental exposures linked to urbanization, pollution, and other structural determinants. Preexisting chronic lung diseases and hereditary cancer syndromes should also be considered, as they may modify risk and clinical evolution [6].

Survival depends largely on early diagnosis. When lung cancer is detected at stage I, survival may exceed 60%-70% in patients who are candidates for appropriate surgical intervention. In addition, the different subtypes of lung cancer, particularly non-small cell lung cancer, require precise histological and molecular characterization to guide individualized therapeutic decisions [6]. Therefore, recognizing these subtypes is crucial, as they differ in terms of histology, clinical behavior, and treatment approach.

Molecular biomarker testing has become a fundamental component of the individualized approach to lung cancer [6,7]. These markers allow a better understanding of tumor biology and support the selection of targeted therapies or immunotherapy in eligible patients. Thus, the challenge for health systems is not limited to early detection but also includes ensuring timely access to molecular testing and treatments based on the clinical and biological characteristics of the tumor.

Clinical guidelines recommend processes for oncological care and their corresponding timeframes [8-16]. Nevertheless, significant delays persist in both diagnosis and treatment initiation, highlighting the need for a comprehensive approach that considers individual patient characteristics and cancer stage.

In this context, this study aimed to describe the clinical, sociodemographic, and epidemiological characteristics of adults with primary lung cancer treated at Hospital México of the Caja Costarricense de Seguro Social (CCSS) between 2015 and 2020; to assess diagnostic and therapeutic care pathway intervals, including the intervals from documented suspicion to pathological diagnosis, from referral to first care episode, from diagnosis to first treatment, and from first care episode to death; and to describe molecular biomarker testing, access to targeted therapy or immunotherapy, and survival from the first care episode related to suspected lung cancer.

The CCSS is the main public health care provider in Costa Rica [17]. Within its network, three national hospitals operate at the tertiary level of care. Among them, Hospital México serves the northwestern network, with an approximate direct or indirect coverage of 2 million people, including patients from the Greater Metropolitan Area and rural regions such as the Central Pacific and Guanacaste, mainly referred from regional and peripheral hospitals at the secondary level of care.

## Materials and methods

We conducted a retrospective observational study with a quantitative approach based on medical record review. Patients older than 18 years with a pathological diagnosis of primary lung cancer treated at Hospital México, a public tertiary-level hospital of the CCSS in Costa Rica, were included. The initial population was identified from the Pathology Department database for the 2015-2020 period (n=278). Physical medical records could not be accessed for 18 patients. After medical record review, 37 records were excluded because they did not meet the inclusion criterion of a pathological diagnosis of primary lung cancer. The final analytic cohort included 223 patients. Figure 1 summarizes the patient selection process.

**Figure 1:**
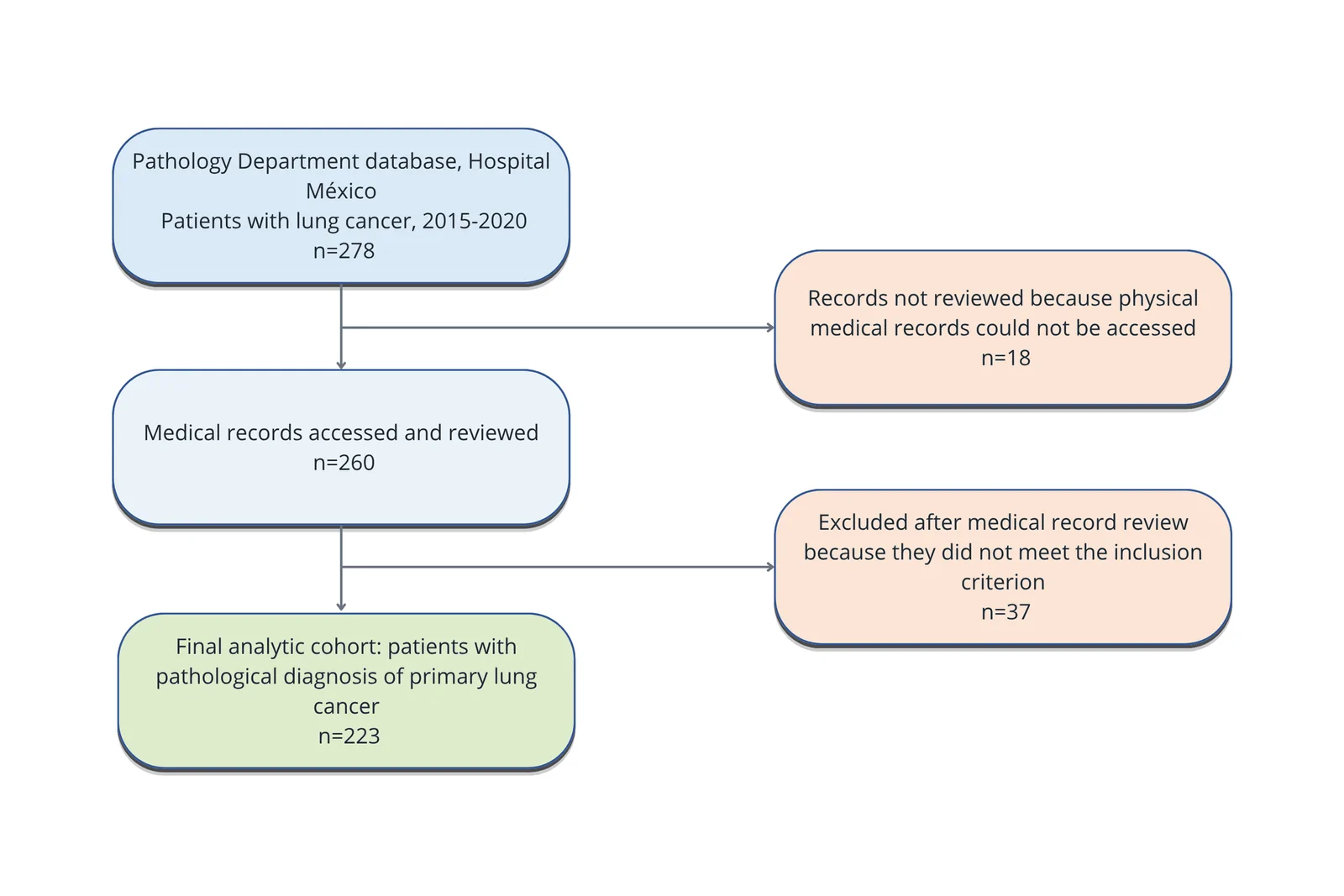
Flowchart of patient selection for the analytic cohort of patients with primary lung cancer treated at Hospital México, Costa Rica, 2015-2020 Physical medical records could not be accessed for 18 patients. Image credits: The figure was manually created by the authors using Canva (Canva Pty Ltd., Sydney, Australia).

Physical medical records were reviewed using a standardized instrument that included the following variables: diagnosis of primary lung cancer, lesion location, histological type, tumor stage, smoking history, molecular biomarkers, comorbidities, vital status, time between documented suspicion and pathological diagnosis, symptoms before diagnosis, history of tuberculosis, and sociodemographic variables, among others.

The instrument was completed only within Hospital México to protect data confidentiality. Each medical record was reviewed twice to maximize the completeness and consistency of the information collected.

For the purpose of this study, documented suspicion of lung cancer was operationalized as the earliest dated event available in the Hospital México medical record that was related to the diagnostic work-up for suspected lung cancer. This definition depended on the information available in the tertiary-level medical record and did not necessarily capture symptom onset, first contact with primary care, or clinical events that occurred before referral to Hospital México.

The first care episode related to suspected lung cancer was defined as the earliest dated care pathway event recorded in the medical record and linked to the evaluation of suspected lung cancer. Pathological diagnosis was defined as the date of the pathology report confirming primary lung cancer. The first treatment date was defined as the first recorded date of oncological treatment, including surgery, radiotherapy, chemotherapy, targeted therapy, or immunotherapy, according to the information available in the medical record.

For clinical variables recorded as “present” or “ruled out,” percentages were calculated using the subtotal of patients with available information for that variable as the denominator, defined as the sum of cases with a recorded finding and cases in which the finding was ruled out. Patients without available records for a specific variable were not included in that variable-specific denominator. When information was explicitly unavailable or not recorded, this was retained as missing or not recorded, as appropriate. No imputation of missing values was performed. Therefore, denominators varied across variables according to data availability in the medical records.

Univariate and bivariate descriptive analyses were performed according to the nature of the variables. For quantitative variables, normality was assessed using the Shapiro-Wilk test. According to the observed distribution, results were presented as mean and SD or as median and IQR. Categorical variables were described using absolute frequencies and percentages.

For bivariate analysis, the chi-square test, Fisher’s exact test, the Mann-Whitney U test, and Student’s t-test were used as appropriate. When applicable, test statistics were reported as χ² for Pearson’s chi-square test, t for Student’s t-test, z for the Mann-Whitney U test, and W for the Shapiro-Wilk test. Fisher’s exact tests were reported using exact p-values. Survival was estimated using the Kaplan-Meier method. The time origin for survival analysis was the first care episode related to suspected lung cancer, not the date of pathological diagnosis. The event was death documented during follow-up. Patients without documented death in the available records were treated as censored at the end of follow-up or at the last available record, according to the information available in the medical record. A formal loss-to-follow-up rate could not be reliably estimated from the retrospective medical record data. Therefore, survival estimates should be interpreted as survival from the first care episode related to suspected lung cancer. A statistical significance level of 5% was used. Analyses were performed using Stata Statistical Software, version 14 (StataCorp, College Station, TX, USA). Bivariate analyses were interpreted as exploratory. No multivariable model was performed; therefore, associations should not be interpreted as causal or as independently adjusted prognostic effects.

Molecular biomarker testing was considered present when at least one molecular or immunohistochemical biomarker report was documented in the medical record. The specific biomarkers recorded included those explicitly reported in the chart. The study did not aim to evaluate the technical methodology of biomarker testing; therefore, information on whether testing was performed using PCR, immunohistochemistry, next-generation sequencing, or another platform was recorded only when available and was not used as an analytical criterion.

Access to targeted therapy or immunotherapy was defined as documentation in the medical record that the patient received at least one targeted agent or immune checkpoint inhibitor. For treatment-related analyses, percentages were calculated using the subset of patients with oncological treatment recorded as the denominator, unless otherwise specified. Administrative requests to higher institutional authorities outside the standard care protocol were described among patients who received targeted therapy or immunotherapy. Patients who received chemotherapy without targeted therapy or immunotherapy were not treated as a formal comparator group for access analyses; treatment categories were described according to the information documented in the medical record.

The study was approved by the Scientific Ethics Committee of Hospital México of the CCSS through approval letter CEC-HM-0097-2023. As this was a retrospective medical record review, the committee waived informed consent. The database was anonymized and did not include information that could identify individual patients.

## Results

In the analyzed population, female patients represented the largest proportion of the cohort (n=122; 54.71%). In the bivariate analysis, male sex was associated with a higher probability of mortality compared with female sex (OR: 6.71; 95% CI: 1.48-30.34). The median age of patients who were alive was 73 years (IQR: 68-82), whereas the median age of patients who had died was 66 years (IQR: 59-78) (Table 1).

**Table 1:** Absolute and percentage distribution of the sociodemographic characteristics of patients with primary lung cancer according to mortality at a public hospital in Costa Rica *Chi-square test. **Fisher’s exact test. Note: Percentages were calculated based on the number of patients with available information for each sociodemographic variable. Statistical tests: Pearson’s chi-square test was used for sex: χ²(1)=7.889; p=0.005. Fisher’s exact test was used for age, province, education, occupation, nationality, and marital status: age, exact p=1.000; province, exact p=0.553; education, exact p=0.266; occupation, exact p=0.029; nationality, exact p=0.357; marital status, exact p=0.099.

Sociodemographic profile	Total		Mortality +		p-value
	n	%	n	%	
Sex					
Female	122	54.71	97	79.51*	0.005
Male	101	45.29	93	92.08	
Age					
<60 years	54	24.77	50	92.59**	1.000
≥60 years	164	75.23	137	83.54	
Province					
Alajuela	85	38.64	74	87.06**	0.553
San José	41	18.64	32	78.05	
Guanacaste	34	15.45	32	94.12	
Puntarenas	32	14.55	26	81.25	
Heredia	28	12.73	23	82.14	
Education					
Less than complete primary education	16	25.81	14	87.50**	0.266
Complete primary education	13	20.97	11	84.62	
Incomplete secondary education	7	11.29	4	57.14	
Complete secondary education	2	3.23	2	100.00	
More than complete secondary education	24	38.71	20	83.33	
Occupation					
Worker, formal or informal	78	45.61	70	89.74**	0.029
Retired	56	32.75	51	91.07	
Homemaker	34	19.88	29	85.29	
Not working	3	1.75	1	33.33	
Nationality					
Costa Rican	199	91.71	181	90.95**	0.357
Nicaraguan	11	5.07	3	27.27	
Other	7	3.23	2	28.57	
Marital status					
Married	110	52.13	97	88.18**	0.099
Single	42	19.91	31	73.81	
Widowed	25	11.85	25	100.00	
Divorced	20	9.47	18	90.00	
Common-law union	14	6.64	12	85.71	

The most frequent province of residence was Alajuela, followed by San José and Guanacaste. Most patients were Costa Rican (n=199; 91.71%), followed by patients of Nicaraguan nationality (n=11; 5.07%). More than half were married or in a common-law union (n=124; 58.77%), and a substantial proportion had not completed secondary education (n=36; 58.06%). Regarding occupation, 78 patients (45.61%) were formal or informal workers, including farmers, merchants, construction workers, drivers, cooks, office workers, fishermen, teachers, and mechanics, among others.

Table 2 shows that, at the close of follow-up in 2025, 190 patients (85.20%) had died (95% CI: 79.87%-89.31%). Table 3 shows that 35 patients (15.70%) had a history of another primary cancer, with breast cancer and melanoma being the most frequent.

**Table 2:** Absolute and percentage distribution of mortality among patients with primary lung cancer at a public hospital in Costa Rica Note: Percentages were calculated based on the total number of patients included in the cohort.

Mortality	n	%	95% CI	
Yes	190	85.20	79.87	89.31
No	16	7.17	4.78	11.96
Not recorded	17	7.62	4.42	11.43

**Table 3:** Absolute and percentage distribution of other types of cancer reported in patients with primary lung cancer at a public hospital in Costa Rica Note: The percentage of “other reported cancer” was calculated based on the total cohort. Percentages by type of cancer were calculated based on the total number of patients with a history of another primary cancer. CNS, central nervous system

Clinical history	Total		Mortality +		
	n	%	n	%	
Other reported cancer					
Yes	35	15.70	27	77.14	
Type of reported cancer					
Breast	8	22.86	4	50.00	
Melanoma	6	17.14	4	66.67	
Colon	4	11.43	4	100.00	
Prostate	4	11.43	3	75.00	
CNS	2	5.71	1	50.00	
Other	11	31.43	11	100.00	

Regarding smoking, 97 patients (50.00% of those with available smoking status) currently smoked or had smoked at some point in their lives. Among those with a history of smoking, the median age at initiation was 15 years (IQR: 12-20). Among those who had stopped smoking, the mean age at cessation was 56.53 years (SD: 11.17), which could be related to age at diagnosis. The mean duration of smoking was 39.77 years (SD: 15.20) (Table 4).

**Table 4:** Absolute and percentage distribution of smoking, drug use, and other exposures reported in the medical records of patients with primary lung cancer at a public hospital in Costa Rica *Shapiro-Wilk, p<0.05 **Chi-square test/Fisher’s exact test ***Mann-Whitney U test ****Student’s t-test Note: Percentages were calculated based on the total number of patients with available information for each variable. For variables with “recorded” and “ruled out” categories, the denominator corresponded to the sum of both categories. Statistical tests: Normality was assessed using the Shapiro-Wilk test: age at smoking initiation, W=0.921; p=0.002; age at smoking cessation, W=0.984; p=0.779; years of smoking, W=0.979; p=0.609; number of cigarette packs per week, W=0.870; p<0.001. For comparisons according to mortality, Fisher’s exact test was used for smoking status, passive smoking, wood smoke exposure, and exposure to inorganic dusts. Exact p-values were 0.306, 0.658, 0.016, and 0.278, respectively. The Mann-Whitney U test was used for age at smoking initiation and number of cigarette packs per week: z=1.903; p=0.057 and z=-0.757; p=0.449, respectively. Student’s t-test was used for age at smoking cessation and years of smoking: t(41)=-0.514; p=0.610 and t(38)=-1.264; p=0.214, respectively.

Drug use and exposures	Total		Mortality +		p-value
	n	%	n	%	
Smoking status					
Current smoker	32	16.49	29	90.63**	0.306
Former smoker	65	33.51	58	89.23	
Never-smoker	97	50.00	78	80.41	
Age at smoking initiation*					
Median (IQR)	15 (12-20)		15 (12-20)***		0.057
Age at smoking cessation					
Mean (SD)	56.53 (±11.17)		56.85 (±11.33)****		0.610
Years of smoking					
Mean (SD)	39.77 (±15.20)		40.51 (±15.55)****		0.214
Number of cigarette packs per week*					
Median (IQR)	6.05 (1.05-7)		6.05 (1-7)***		0.449
Passive smoking					
Recorded in medical record	21	9.42	18	85.71**	0.658
Other substances					
Alcohol	55	24.66	49	89.09	
Marijuana	1	0.45	1	100.00	
Combination of the above	8	3.59	8	100.00	
No reported substance use	69	30.94	56	81.16	
Wood smoke exposure					
Recorded in medical record	60	26.91	50	83.33**	0.016
Exposure to inorganic dusts					
Recorded in medical record	4	1.79	3	75.00**	0.278

In this same category, a median of 6.05 packs of 20 cigarettes per week was estimated (IQR: 1.05-7). Passive exposure to tobacco smoke was documented in 21 medical records (9.42%); in most of these cases, exposure exceeded 15 years, and exposures longer than 50 years were also reported. Only three medical records specified that the source of exposure was the patient’s partner.

In terms of other substances, alcohol use was the most frequent (n=55; 24.66%). In 10 patients, it was documented as an alcohol-related disease; in the remaining cases, it was recorded as occasional or social use. Four cases of illicit drug use were also reported, including marijuana, crack, and cocaine.

In Table 5, the most frequent comorbidities were arterial hypertension (n=128; 57.40%) and diabetes mellitus (n=43; 19.28%). In addition, chronic obstructive pulmonary disease (COPD) was documented in 20 patients (8.97%), and a history of tuberculosis was documented in four patients (1.79%).

**Table 5:** Absolute and percentage distribution of comorbidities reported in the medical records of patients with primary lung cancer at a public hospital in Costa Rica Note: Percentages were calculated based on the total number of patients with available information for each variable. For variables with “recorded” and “ruled out” categories, the denominator corresponded to the sum of both categories. COPD, chronic obstructive pulmonary disease; TB, tuberculosis; HTN, hypertension; DM, diabetes mellitus

Clinical history	Total		Mortality +	
	n	%	n	%
COPD				
Recorded in medical record	20	8.97	18	69.23
COPD ruled out	6	2.69	4	15.38
TB				
Recorded in medical record	4	1.79	3	5.45
TB ruled out	51	22.87	44	80.00
HTN				
Recorded in medical record	128	57.40	108	84.38
DM				
Recorded in medical record	43	19.28	37	86.05
Asthma				
Recorded in medical record	20	8.97	12	60.00
Heart disease				
Recorded in medical record	20	8.97	18	90.00
Hypothyroidism				
Recorded in medical record	16	7.17	14	87.50
Obesity				
Recorded in medical record	9	4.04	6	66.67

The most frequent signs and symptoms before diagnosis were cough (n=126; 56.50%), mainly dry cough, followed by dyspnea (n=110; 49.33%), weight loss (n=98; 43.95%), pleural effusion (n=79; 35.43%), with a predominance on the right side, chest pain (n=57; 25.56%), and hemoptysis (n=24; 10.76%) (Table 6).

**Table 6:** Absolute and percentage distribution of signs and symptoms reported in the medical records of patients with primary lung cancer at a public hospital in Costa Rica Note: Percentages were calculated based on the total number of patients with available information for each variable. For variables with “recorded” and “ruled out” categories, the denominator corresponded to the sum of both categories.

Signs and symptoms before diagnosis	Total		Mortality +	
	n	%	n	%
Cough				
Recorded in medical record	126	56.50	107	84.92
Dyspnea				
Recorded in medical record	110	49.33	93	84.55
Weight loss				
Recorded in medical record	98	43.95	84	85.71
Pleural effusion				
Recorded in medical record	79	35.43	72	91.14
Left	23	10.31	21	26.58
Right	36	16.14	34	43.04
Bilateral	5	2.24	4	5.06
Chest pain				
Recorded in medical record	57	25.56	51	89.47
Hemoptysis				
Recorded in medical record	24	10.76	21	87.50
Fever				
Recorded in medical record	16	7.17	13	81.25
Fatigue/asthenia/weakness				
Recorded in medical record	16	7.17	15	93.75
Arm pain				
Recorded in medical record	14	6.28	12	85.71
Hyporexia				
Recorded in medical record	13	5.83	12	92.31
Vomiting/nausea				
Recorded in medical record	11	4.93	11	100.00
Ataxia/gait problems				
Recorded in medical record	9	4.04	9	100.00
Atelectasis				
Recorded in medical record	8	3.59	7	87.50
Headache/migraine				
Recorded in medical record	8	3.59	7	87.50
Bronchophony/dysphonia				
Recorded in medical record	5	2.24	5	100.00
Sleep disturbance				
Recorded in medical record	6	2.69	4	66.67
Diaphragmatic/vocal cord paralysis				
Recorded in medical record	4	1.79	4	100.00
Paraneoplastic syndrome				
Recorded in medical record	3	1.35	3	100.00
Hemiparesis				
Recorded in medical record	3	1.35	3	100.00
Seizures				
Recorded in medical record	3	1.35	3	100.00
Dysphagia				
Recorded in medical record	4	1.79	4	100.00
General malaise				
Recorded in medical record	2	0.90	2	100.00
Upper-limb paresthesia				
Recorded in medical record	2	0.90	1	50.00
Sweating				
Recorded in medical record	2	0.90	2	100.00
Bronchitis				
Recorded in medical record	2	0.90	2	100.00
Empyema				
Recorded in medical record	1	0.45	1	100.00
Depression/anxiety				
Recorded in medical record	1	0.45	1	100.00
Wheezing				
Recorded in medical record	1	0.45	1	100.00

Regarding abdominal or thoracic CT scans performed before diagnosis, at least one CT scan was documented in 184 patients (82.51%), with a minimum of one and a maximum of six per patient. For the first CT scan with available information on the date of the study and the report, the median time between the study and the report was eight days (IQR: 2-17). Most of these studies were performed at CCSS health care centers (Table 7).

**Table 7:** Absolute and percentage distribution of diagnostic testing: CT scans reported before diagnosis in the medical records of patients with primary lung cancer at a public hospital in Costa Rica *Shapiro-Wilk test, p<0.05 **Shapiro-Wilk test, p>0.05 Note: Percentages were calculated based on the subtotal of patients with available information for each variable, defined as recorded positive findings plus ruled-out findings. Statistical tests: Normality was assessed using the Shapiro-Wilk test: number of CT scans, W=0.868; p<0.001; time between CT scan 1 and report, W=0.250; p<0.001; overall time between CT scans and report, W=0.248; p<0.001. For comparisons according to mortality, the Mann-Whitney U test was used: number of CT scans, z=0.667; p=0.505; time between CT scan 1 and report, z=0.797; p=0.426; overall time between CT scans and report, z=0.588; p=0.557.

CT	Total		Mortality +		p-value
	n	%	n	%	
CT: abdomen/chest					
Number of patients with CT scans recorded	184	82.51	155	84.24	
Number of CT scans, median (IQR)*	1 (1-2)		1 (1-2)		0.505
First abdominal/chest CT scan (n=57)					
Time between CT scan 1 and report*, median (IQR)**	8 (2-17)		8 (3-17)		0.426
Health care center					
Hospital México	85	46.20			
Regional hospital	53	28.80			
Private center	23	12.50			
Peripheral hospital	12	6.52			
Specialized hospital	2	1.09			
Health areas/cooperatives	1	0.54			
Unknown	8	4.35			
All abdominal/chest CT scans					
Overall time between CT scans and report, median (IQR)*	7.75 (3-29)		7.5 (3-29.5)		0.557
CT: neck					
Number of patients with CT scans recorded	1	0.45	1	100.00	
C: head/brain					
Number of patients with CT scans recorded	28	12.56	27	96.43	

In Table 8, at least one chest or abdominal radiograph was documented in 55 patients (24.66%), and at least one chest or abdominal MRI study was documented in six patients (2.69%). For MRI, the mean time between the study and the report was 8.67 days (SD: 10.79). In addition, 154 patients (69.06%) had at least one bronchoscopy recorded.

**Table 8:** Absolute and percentage distribution of other diagnostic tests reported before diagnosis in the medical records of patients with primary lung cancer at a public hospital in Costa Rica *Variable summarized as median (IQR). Note: Percentages were calculated based on the subtotal of patients with available information for each variable, defined as recorded positive findings plus ruled-out findings. Statistical tests: Normality was assessed using the Shapiro-Wilk test: number of X-rays, W=0.519; p<0.001; number of bronchoscopy/endoscopy procedures, W=0.895; p<0.001. For comparisons according to mortality, the Mann-Whitney U test was used: number of X-rays, z=-0.253; p=0.801; number of bronchoscopy/endoscopy procedures, z=0.158; p=0.875.

Other diagnostic tests	Total		Mortality +		p-value
	n	%	n	%	
Radiography: chest, abdomen					
Number of patients with X-rays recorded	55	24.66	47	85.45	
Number of X-rays, median (IQR)*	1 (1-1)		1 (1-1)		0.801
1	54	98.18	46	83.64	
2	1	1.82	1	1.82	
Time between X-ray and report	Not recorded				
Health care center					
Hospital México	24	43.64			
Regional hospital	13	23.64			
Private center	8	14.55			
Peripheral hospital	4	7.27			
Health areas/cooperatives	3	5.45			
Unknown	3	5.45			
MRI: chest, abdomen					
Number of patients with MRI recorded	6	2.69	5	83.33	
Number of MRI studies	1 (1-1)				
Time between MRI and report	8.67 (±10.79)				
Health care center					
Hospital México	5	83.33			
Regional hospital	1	16.67			
Bronchoscopy/endoscopy: lungs					
Number of patients with procedures recorded	154	69.06	133	86.36	
Number of bronchoscopy/endoscopy procedures, median (IQR)*	1 (1-1)		1 (1-1)		0.875
1	122	79.22	107	69.48	
2	27	17.53	23	14.94	
3	5	3.25	3	1.95	
Health care center					
Hospital México	131	86.75	113	74.83	
Regional hospital	17	11.26	14	9.27	
Specialized hospital	2	1.32	2	1.32	
Peripheral hospital	1	0.66	1	0.66	

With respect to biopsies performed before the definitive diagnosis, at least one biopsy was documented in 218 patients (97.76%). The median was two biopsies per patient, with a minimum of one and a maximum of eight. In 12 patients with biopsies (5.50%), the pathology result became available after the patient had died. Considering all biopsy-to-report intervals per patient, the median was 15 days, with a minimum of three and a maximum of 244 days (Table 9).

**Table 9:** Absolute and percentage distribution of prediagnostic lung biopsy reports in the medical records of patients with primary lung cancer at a public hospital in Costa Rica Note: Percentages were calculated based on the subtotal of patients with available information for each variable, defined as recorded positive findings plus ruled-out findings. Statistical tests: Normality was assessed using the Shapiro-Wilk test: number of prediagnostic biopsies, W=0.907; p<0.001; overall biopsy-to-report time, W=0.255; p<0.001. For comparisons according to mortality, the Mann-Whitney U test was used: number of prediagnostic biopsies, z=0.035; p=0.972; overall biopsy-to-report time, z=1.394; p=0.163.

Prediagnostic lung biopsy reports	Total		Mortality +			p-value
	n	%	n		%	
Prediagnostic biopsies						
Number of patients with biopsies recorded	218	97.76	187	85.78		
Number of biopsies, median (IQR)	2 (1-2)		2 (1-3)			0.972
Deaths before pathology result	12	5.50	-			
Time between biopsy 1 and report (n=209), median (IQR)	13 (9-20)					
Time between biopsy 2 and report (n=131), median (IQR)	13 (7-19)					
Time between biopsy 3 and report (n=53), median (IQR)	11 (7-18)					
Time between biopsy 4 and report (n=17), median (IQR)	12 (9-21)					
Time between biopsy 5 and report (n=6), median (IQR)	17 (8-34)					
Time between biopsy 6 and report (n=2), median (IQR)	11 (7-15)					
Time between biopsy 7 and report (n=1), median (IQR)	26 (26-26)					
Time between biopsy 8 and report (n=1), median (IQR)	6 (6-6)					
Overall biopsy-to-report time (n=131), median (IQR)	15 (9-21)					0.163

Post-diagnostic biopsies were documented in 71 patients (31.84%). Molecular biomarker testing was recorded in 106 patients (47.53%). Among the reported biomarkers, EGFR, ALK, and PD-L1 predominated. Information on the center where the test was performed was available for a subset of reports, of which 30 reports (60.00%) came from national hospitals within the CCSS (Table 10).

**Table 10:** Absolute and percentage distribution of biomarker reports in the medical records of patients with primary lung cancer at a public hospital in Costa Rica Note: Percentages were calculated based on the subtotal of patients with available information for each variable, defined as recorded positive findings plus ruled-out findings. The specific biomarkers reported do not necessarily correspond to mutually exclusive categories. Counts for individual biomarkers correspond to biomarkers explicitly named in available reports and may not sum to the total number of patients with any biomarker report. Information on the center where the test was performed was available for a subset of reports. HCG, Hospital Dr. Rafael Ángel Calderón Guardia; HSJD, Hospital San Juan de Dios

Recorded test	Total		Mortality +	
	n	%	n	%
Post-diagnostic biopsies				
Number of patients with biopsies recorded	71	31.84	54	76.06
Number of biopsies, median (IQR)	2 (1-3)		2 (1-3)	
Biomarkers				
Number of patients with biomarkers recorded	106	47.53	91	85.85
EGFR	40	37.74	30	75.00
BRAF	1	0.94	1	100.00
ALK	9	8.49	8	88.89
PD-L1	5	4.72	5	100.00
TP53	1	0.94	1	100.00
NF1	1	0.94	1	100.00
T790M	3	2.83	3	100.00
Health care center				
Public center (HCG and HSJD)	30	60.00	24	48.00
Private center	20	40.00	17	34.00

The immunohistochemistry report was available in 193 cases (86.55%). The most frequent positive markers were CK7, TTF-1, Napsin A, PAN CK, synaptophysin, and P63. According to the medical record review, 113 tumors (58.25%) were located on the right side, and the most frequent location was the upper lobe (n=74; 46.84%). The predominant histological type was adenocarcinoma (n=194; 91.51%), and the most frequent degree of differentiation was poorly differentiated (n=80; 45.45%). Regarding TNM (Tumor, Node, Metastasis) staging, T4 was the most frequent tumor category (n=70; 50.00%), N3 was reported in 41 patients (29.29%), and M1a in 45 patients (31.91%), suggesting a substantial proportion of patients with characteristics compatible with advanced disease. Formal American Joint Committee on Cancer (AJCC) stage groups were not reconstructed because complete clinical or pathological grouping information was not consistently available in the medical record (Table 11).

**Table 11:** Absolute and percentage distribution of lung cancer location and histology in patients with primary lung cancer at a public hospital in Costa Rica Note: Percentages were calculated based on the subtotal of patients with available information for each variable, defined as recorded positive findings plus ruled-out findings.

Lung cancer	Total		Mortality +	
	n	%	n	%
Location (topography)				
Lower lobe	58	36.71	51	87.93
Middle lobe	10	6.33	9	90.00
Upper lobe	74	46.84	58	78.38
Upper and middle lobes	6	3.80	4	66.67
Lower and middle lobes	8	5.06	6	75.00
Lower and upper lobes	2	1.27	1	50.00
Laterality				
Left	76	39.18	66	86.84
Right	113	58.25	90	79.65
Bilateral	5	2.58	5	100.00
Histological type (morphology)				
Adenocarcinoma	194	91.51	167	86.08
Neuroendocrine tumor	6	2.83	1	16.67
Small-cell carcinoma	1	0.47	1	100.00
Squamous cell carcinoma	5	2.36	4	80.00
Adenocarcinoma + small-cell carcinoma + neuroendocrine tumor	1	0.47	1	100.00
Small-cell carcinoma + neuroendocrine tumor	2	0.94	2	100.00
Adenocarcinoma + small-cell carcinoma	2	0.94	2	100.00
Other	1	0.47	1	100.00
Differentiation				
Poorly differentiated	80	45.45	75	93.75
Moderately differentiated	45	25.57	37	82.22
Well differentiated	51	28.98	41	80.39
Staging				
(T) Tumor				
1	4	2.86	2	50.00
1A	3	2.14	3	100.00
1B	5	3.57	3	60.00
1C	1	0.71	0	0.00
2	10	7.14	9	90.00
2A	9	6.43	7	77.78
2B	9	6.43	7	77.78
3	26	18.57	23	88.46
4	70	50.00	60	85.71
X	3	2.14	3	100.00
(N) Lymph nodes				
0	23	16.43	14	60.87
1	21	15.00	17	80.95
2	45	32.14	41	91.11
2B	1	0.71	1	100.00
3	41	29.29	37	90.24
X	9	6.43	7	77.78
(M) Metastasis				
0	26	18.44	17	65.38
1	22	15.60	19	86.36
1A	45	31.91	40	88.89
1B	21	14.89	19	90.48
1C	6	4.26	6	100.00
2	1	0.71	1	100.00
X	20	14.18	16	80.00

Regarding the oncological treatment recorded, 150 patients (67.26%) received some type of treatment. A total of nine patients (4.04%) were not candidates for treatment due to tumor characteristics, and palliative management was therefore recommended. Chemotherapy was the most frequent treatment, recorded in 83 patients (55.33%), followed by radiotherapy in 58 patients (38.67%). Targeted therapy or immunotherapy was documented in 45 patients (30.00%); within this group, erlotinib was the most frequently reported medication (Table 12).

**Table 12:** Absolute and percentage distribution of treatment among patients with primary lung cancer at a public hospital in Costa Rica *Variable summarized as median (IQ). Note: Percentages were calculated based on the subtotal of patients with available information for each variable, defined as recorded positive findings plus ruled-out findings. Statistical tests: For comparisons according to mortality, Fisher’s exact test was used for any treatment recorded and type of surgery: exact p=0.601 and exact p=0.007, respectively. Normality was assessed using the Shapiro-Wilk test: number of RT sessions, W=0.781; p=0.002; time between first RT report and final RT session, W=0.713; p<0.001; time between start and end of targeted therapy/immunotherapy, W=0.927; p=0.040; time between start and end of chemotherapy, W=0.600; p<0.001. For continuous treatment-time comparisons according to mortality, the Mann-Whitney U test was used: number of RT sessions, z=-1.629; p=0.103; time between first RT report and final RT session, z=-0.048; p=0.962; time between start and end of targeted therapy/immunotherapy, z=1.444; p=0.149; time between start and end of chemotherapy, z=0.974; p=0.330. RT, radiotherapy

Treatment	Total		Mortality +		p-value
	n	%	n	%	
Any treatment recorded	150	67.26	123	82.00	0.601
Not eligible for treatment	9	4.04	9	100.00	
Surgery	62	41.70	44	70.97	
Number 1	52	83.87			
Number 2	8	12.90			
Number 3	1	1.61			
Type of surgery					
Lobectomy	10	16.13	6	60.00	0.007
Thoracotomy	10	16.13	9	90.00	
Lobectomy + thoracotomy	6	9.68	3	50.00	
Bilobectomy; lymphadenectomy	1	1.61	0	0.00	
Thoracotomy; lobectomy; lymphadenectomy	2	3.23	0	0.00	
Lobectomy; lymphadenectomy	6	9.68	3	50.00	
Thoracotomy; pulmonary decortication	1	1.61	1	100.00	
Thoracotomy; bilobectomy	1	1.61	1	100.00	
Thoracotomy; lymphadenectomy	1	1.61	1	100.00	
Other	22	35.48	19	86.36	
Not specified	2	3.23			
Radiotherapy					
Any RT appointment recorded	58	38.67	53	91.38	
Not eligible for RT	2	1.33			
Patient declined RT	2	1.33			
Number of sessions, median (IQR)*	6	(5-10)	8	(5-10)	0.103
Time between first RT report and final RT session (n=30), median (IQR)	41	(11-109)	35.5	(11-86)	0.962
Targeted therapy/immunotherapy					
Recorded in medical record	45	30.00	35	77.78	
Report to pharmacovigilance	29	64.44	24	82.76	
Medication (n=48)					
Erlotinib	33	68.75	24	72.73	
Atezolizumab	6	12.50	6	100.00	
Pembrolizumab	3	6.25	3	100.00	
Ceritinib	2	4.17	1	50.00	
Other	4	8.33	3	75.00	
Time between start and end of targeted therapy/immunotherapy (n=30), median (IQR)	275	(125-537)	244	(12-506.5)	0.149
Therapy discontinued due to allergy	2	4.44	1	50.00	
Chemotherapy					
Recorded in medical record	83	55.33	77	92.77	
Not eligible for chemotherapy	13	15.66	12	92.31	
Patient declined chemotherapy	6	7.23	3	50.00	
Discontinued due to toxicity	6	7.23	6	100.00	
Number of sessions, median (IQR)*	4	(4-8)	4	(4-8)	
Time between start and end of chemotherapy (n=80), median (IQR)	63.5	(63-109)	63	(63-965)	0.330
Paclitaxel (Taxol)	72	86.75			
Cisplatin	50	60.24			
Carboplatin	37	44.58			
Docetaxel	11	13.25			
Gemcitabine	2	2.41			
Pemetrexed	2	2.41			
Vinorelbine	1	1.20			

Within the CCSS service network, 124 patients (58.76%) had a referral to Hospital México for evaluation of suspected lung cancer. Most of these referrals came from public health care centers (Table 13).

**Table 13:** Absolute and percentage distribution of the management of suspected primary lung cancer at a public hospital in Costa Rica *Chi-square test **Fisher’s exact test Note: Percentages were calculated based on the subtotal of patients with available information for each variable, defined as recorded positive findings plus ruled-out findings. Statistical tests: For referral to Hospital México, the Pearson chi-square test was used: χ²(1)=0.369; p=0.543. Fisher’s exact test was used for number of referrals, type of referral, referral center, and service referred to: number of referrals, exact p=1.000; type of referral, exact p=0.059; referral center, exact p=0.047; service referred to, exact p=0.908. For referral center, the corresponding Pearson chi-square statistic was χ²(7)=14.035; p=0.051, but Fisher’s exact p-value was reported because of sparse categories. For service referred to, the corresponding Pearson chi-square statistic was χ²(5)=3.326; p=0.650, but Fisher’s exact p-value was reported because of sparse categories. HM, Hospital México

Referrals	Total	Mortality +		p-value
	n	n	%	
Referral to HM for evaluation of suspected lung cancer	124	114	91.94*	0.543
Number of referrals				
1 referral	118	108	91.53**	1.000
2 referrals	4	4	100.00	
Type				
External to HM	110	103	93.64**	0.059
Internal to HM	12	9	75.00	
Referral center				
Regional hospital	58	56	96.55**	0.047
Peripheral hospital	15	15	100.00	
Health area/cooperative	16	12	75.00	
Hospital México	12	10	83.33	
Private center	10	9	90.00	
National hospital other than HM	1	1	100.00	
Specialized hospital	4	4	100.00	
Other	6	6	100.00	
Service referred to				
Pulmonology/neuro-thorax	73	65	89.04**	0.908
Oncology	15	13	86.67	
Emergency department	8	8	100.00	
Radiation oncology	7	7	100.00	
Internal medicine	3	3	100.00	
Other	8	8	100.00	

After diagnosis, the medical records documented care by different services, including oncology, pulmonology, CT, CT angiography, psychology, psychiatry, palliative medicine, and other support services. In addition, 99 patients (44.39%) had at least one hospitalization, with a median of one hospitalization per patient and a median length of stay of 15 days (IQR: 8-31) (Table 14).

**Table 14:** Absolute and percentage distribution of post-diagnostic services and hospitalizations among patients with primary lung cancer at a public hospital in Costa Rica Note: Percentages were calculated based on the subtotal of patients with available information for each variable, defined as recorded positive findings plus ruled-out findings.

Post-diagnostic services	Total		Mortality +	
	n	%	n	%
CT angiography	116	52.02	93	80.17
Median (IQR)	2	(1-4)	2	(1-4)
Nutrition	35	15.70	30	85.71
Median (IQR)	1	(1-2)	1	(1-2)
Oncology, pulmonology, and thoracic surgery	154	69.06	125	81.17
Median (IQR)	3	(1-5)	3	(1-5)
Psychology, psychiatry, and mental health	43	19.28	36	83.72
Median (IQR)	1	(1-2)	1	(1-2)
Social work	13	5.83	11	84.62
Median (IQR)	1	(1-1)	1	(1-1)
Bone scan	33	14.80	29	87.88
Median (IQR)	1	(1-1)	1	(1-1)
Palliative medicine	77	34.53	68	88.31
Median (IQR)	1	(1-2)	1	(1-2)
Radiotherapy	63	28.25	51	80.95
Median (IQR)	1	(1-2)	1	(1-2)
Ultrasound/X-ray	48	21.52	39	81.25
Median (IQR)	1	(1-1)	1	(1-1)
Respiratory therapy	6	2.69	5	83.33
Hospitalizations	99	44.39	81	81.82
Median (IQR)	1	(1-2)	1	(1-2)
Days of hospitalization	15	(8-31)	15	(7.5-30)
Multidisciplinary sessions	42	18.83	38	90.48
Median (IQR)	1	(1-1)	1	(1-1)
Bronchoscopy	30	13.45	24	80.00
Median (IQR)	1	(1-1)	1	(1-1)

In Table 15, the median time between documented suspicion of lung cancer and pathological diagnosis was 34 days (IQR: 21-73). This interval may be underestimated, as it does not necessarily capture the time elapsed between symptom onset, the first general consultation, and communication of the result to the patient by the specialist

**Table 15:** Delays in the suspicion pathway among patients with primary lung cancer at a public hospital in Costa Rica Unit of measurement: days *Shapiro-Wilk test **Mann-Whitney U test for comparison according to mortality. Statistical tests: Normality was assessed using the Shapiro-Wilk test: time between suspicion and diagnosis, W=0.353; p<0.001; time between first referral and first care episode, W=0.327; p<0.001; time between first referral and diagnosis, W=0.435; p<0.001. For comparisons according to mortality, the Mann-Whitney U test was used: time between suspicion and diagnosis, z=2.118; p=0.034; time between first referral and first care episode, z=1.751; p=0.080; time between first referral and diagnosis, z=2.541; p=0.011.

Delays	n	Total					p-value**
		p-value*	Mean	SD	Median	IQR	
Time between suspicion and diagnosis	213	<0.001	92.69	220.19	34	21-73	0.034
Time between first referral and first care episode	89	<0.001	30.27	90.05	6	1-25	0.08
Time between first referral and diagnosis	101	<0.001	78.06	148.53	33	20-70	0.011

In addition, Figure 2 shows high variability in diagnostic times. Although these corresponded to a small number of patients, extreme values exceeding 1000 days were observed. These cases should be interpreted with caution, but they may suggest possible gaps in continuity of care and diagnostic timeliness.

**Figure 2:**
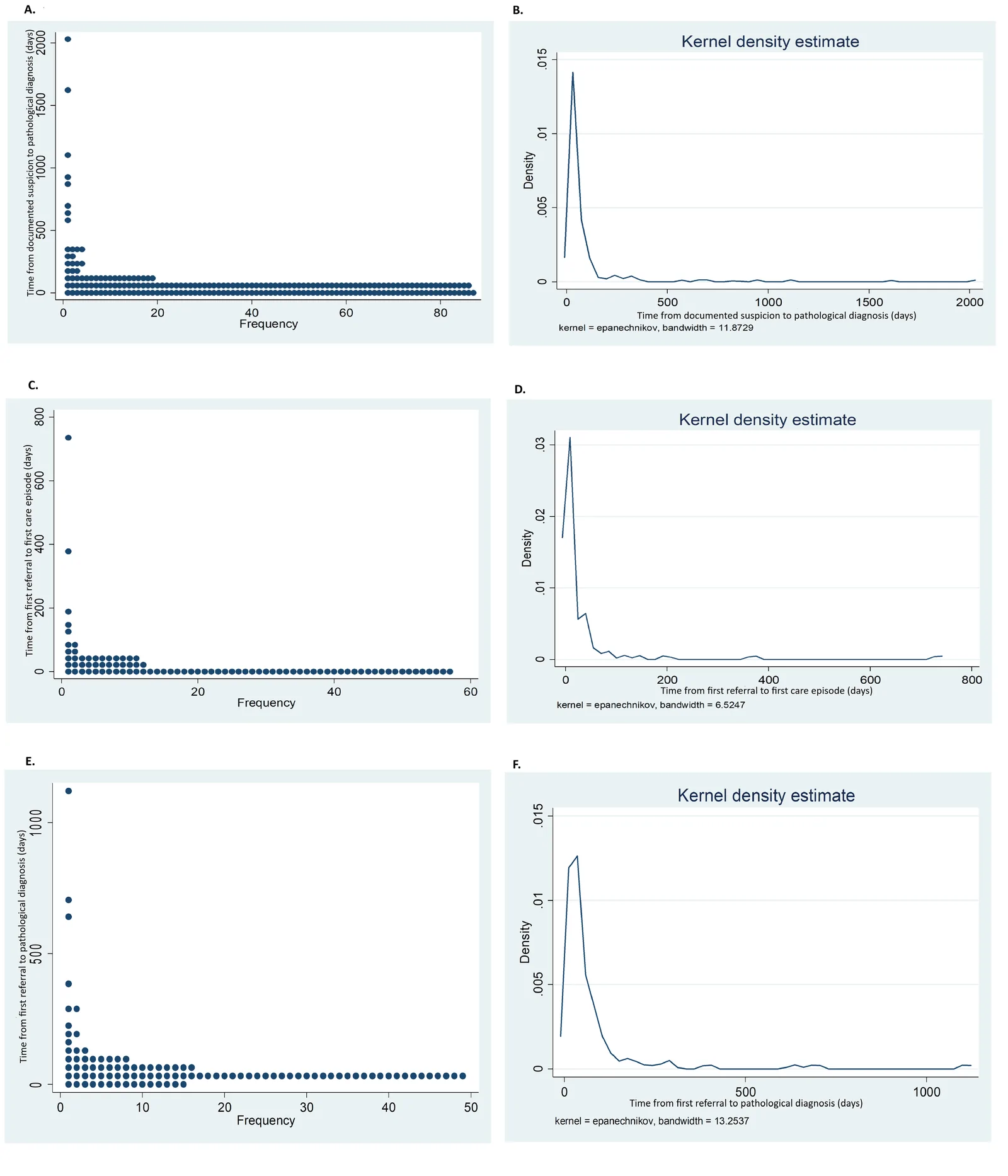
Distribution of diagnostic delay intervals among patients with primary lung cancer treated at a public hospital in Costa Rica A: Frequency distribution of the time from documented suspicion to pathological diagnosis. B: Kernel density estimate of the time from documented suspicion to pathological diagnosis. C: Frequency distribution of the time from first referral to first care episode. D: Kernel density estimate of the time from first referral to first care episode. E: Frequency distribution of the time from first referral to pathological diagnosis. F: Kernel density estimate of the time from first referral to pathological diagnosis. All time intervals are expressed in days.

In Table 16, the median time between diagnosis and first treatment was 46 days (IQR: 29-81). In turn, the median time between the first care episode related to suspicion and death was 188 days (IQR: 71-476).

**Table 16:** Other delays in the suspicion pathway among patients with primary lung cancer at a public hospital in Costa Rica Unit of measurement: days *Shapiro-Wilk test **Mann-Whitney U test for comparison according to mortality. Statistical tests: Normality was assessed using the Shapiro-Wilk test: time between diagnosis and first treatment, W=0.498; p<0.001; time between diagnosis and death, W=0.770; p=0.003; time between first care episode and death, W=0.738; p<0.001; time between first referral and death, W=0.698; p<0.001. For comparisons according to mortality, the Mann-Whitney U test was used when two comparison groups were available: time between diagnosis and first treatment, z=1.437; p=0.151; time between first care episode and death, z=-1.721; p=0.085. Comparisons according to mortality were not calculated for time between diagnosis and death or time between first referral and death because only one comparison group was available in the Stata output.

Delays	n	Total					p-value**
		p-value*	Mean	SD	Median	IQR	
Time between diagnosis and first treatment	117	<0.001	72.98	101.73	46	29-81	0.1509
Time between diagnosis and death	13	<0.001	379.55	461.16	205	72-497	-
Time between first care episode and death	158	0.003	386.25	474.57	188	71-476	0.0852
Time between first referral and death	89	<0.001	411.44	498.75	213	99-494	-

Based on the IQR of survival time, early mortality was concentrated within the first months after the first care episode related to suspected lung cancer, while the upper quartile was reached within approximately one and a half years. The estimated five-year survival from the first care episode related to suspected lung cancer was 1.9% (95% CI: 0.5-5.1). The Kaplan-Meier curves show low survival, with most deaths concentrated within the first one to three years, suggesting that a substantial proportion of patients may have entered the care pathway with characteristics compatible with advanced disease (Figure 3).

**Figure 3:**
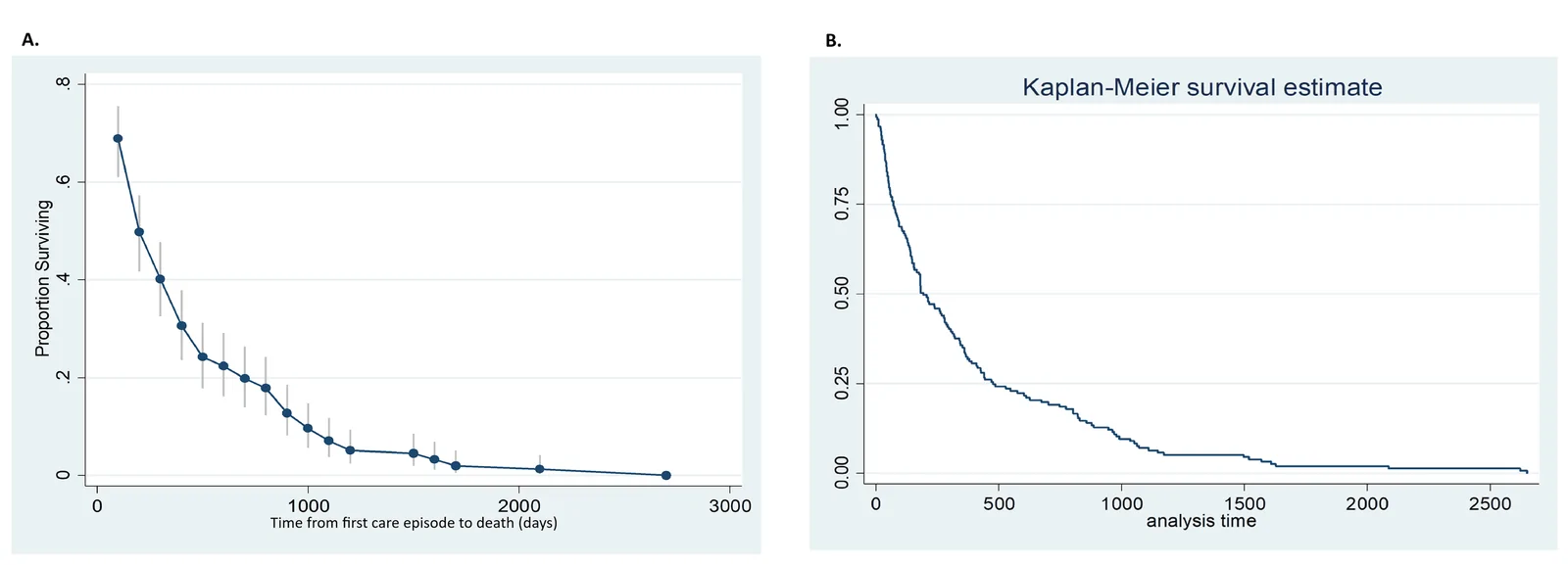
Survival analysis from the first care episode related to suspected lung cancer among patients with primary lung cancer treated at a public hospital in Costa Rica A: Proportion surviving over time with confidence intervals. B: Kaplan-Meier survival estimate. Time is expressed in days.

## Discussion

Lung cancer represents one of the leading causes of cancer-related death in Costa Rica, with increasing mortality despite a downward trend in incidence since 2000. This divergence could be explained, at least in part, by public policy efforts aimed at tobacco control and the prevention of risk factors. However, it could also be influenced by deaths without histological confirmation or by limited access to diagnostic testing in some contexts [18].

Among incident lung cancer cases reported in Costa Rica between 1990 and 2000, higher proportions were observed in men than in women [18]. However, recent studies have described changes in the epidemiological patterns of lung cancer, including a decrease in incidence among men and a higher relative incidence among women in different population groups [19]. Consistent with this, other authors have noted that, in recent decades, the epidemiology of lung cancer has changed and that incidence among women may now be comparable to or even higher than that observed among men [20,21]. In this context, it is necessary to better understand the biological, environmental, and social factors that may influence the development and progression of lung cancer in women.

In the bivariate analysis, men in this study had a higher probability of death compared with women. This finding should be interpreted cautiously because no multivariable adjustment was performed for potential confounders such as age, histological subtype, disease extent, treatment eligibility, or comorbidities. Nevertheless, sex-based differences in lung cancer outcomes have been described in the literature. Li et al. (2025) reported prognostic differences between men and women with non-small cell lung cancer [22], while May et al. (2023) described biological factors, including sex hormones and immune responses, that may contribute to differences in clinical evolution and response to therapy [23].

The present study showed that 190 patients (85.20%) had died by the close of follow-up, reflecting markedly low survival. Based on the IQR of survival time, early mortality was concentrated within the first months after the first care episode related to suspicion, while the upper quartile was reached within approximately one and a half years. These findings are consistent with the literature, which reports five-year survival close to 10%-20% in lung cancer [6], and reinforce that this malignancy remains one of the cancers with the poorest prognosis after diagnosis [3].

Cancer survival is closely related to stage at diagnosis, timely access to the health care system, and treatment availability. Social determinants of health are also increasingly recognized as factors that influence oncological prognosis, particularly when delayed diagnoses and limited access to advanced treatments occur in vulnerable populations [24]. In this sense, the results of this study should be interpreted not only from a clinical perspective but also from a perspective of equity and social justice.

This is consistent with the findings of this cohort, in which a considerable proportion of patients had characteristics compatible with advanced disease, particularly in terms of tumor size and extent. One study identified that tumors measuring 2 cm or more are associated with poorer survival in patients with non-small cell lung cancer [25]. Likewise, survival in this subtype of cancer has been described as being related to disease stage and access to treatments such as radiotherapy or chemotherapy [26].

Regarding treatment, it is relevant that nine patients (4.04%) were not candidates for treatment with curative intent and received a recommendation for palliative care. This finding, together with high mortality and low survival from the first care episode related to suspected lung cancer, is compatible with the possibility that some patients entered the care pathway with advanced disease characteristics. In this scenario, a comprehensive approach is required to strengthen the first level of care, implement clinical algorithms, ensure timely referral, and promote technical coordination through multidisciplinary teams within integrated health service networks. In addition, the results support the need to discuss early detection strategies and assess the feasibility of screening programs directed at at-risk populations as a secondary prevention measure aimed at identifying patients at early stages.

Another relevant finding is that, in 12 patients with biopsies (5.50%), the pathology results became available after death. Rather than indicating an absolute absence of clinical suspicion, this finding shows that some patients died before having definitive histopathological confirmation. In this regard, a systematic review found that a significant proportion of serious diseases were not diagnosed during the patient’s lifetime, including malignant neoplasms [20]. In this cohort, this phenomenon could be related to nonspecific clinical presentation, advanced disease, and delays in the diagnostic process.

The main symptoms reported in this study were cough, dyspnea, and weight loss. These findings are consistent with the literature; however, these symptoms are often nonspecific or mild in the early stages, which may delay health care seeking and, consequently, diagnosis [27-29]. In addition, these symptoms may be confused with respiratory infections, pneumonia, COPD, or asthma exacerbations [30], underscoring the need for greater clinical acuity to raise suspicion of lung cancer earlier.

In this sense, a particularly relevant finding was that a substantial proportion of patients who presented with cough were never-smokers. Therefore, the absence of a smoking history may reduce initial clinical suspicion of lung cancer and contribute to delays between symptom onset, diagnostic testing, and pathological confirmation.

Among patients who reported current or previous tobacco use, age at smoking initiation, age at cessation, years of smoking, and number of cigarette packs per week were not statistically associated with mortality in the bivariate analysis. However, these variables remain clinically relevant given the established relationship between tobacco exposure, cumulative smoking history, and lung cancer prognosis [31-33].

On the other hand, 106 patients (47.53%) in this cohort had access to molecular biomarker testing. This proportion is similar to that reported in a study conducted in Texas, United States, where access was 42.9% [34]; in another study from the same country, only 22% of patients with non-small cell lung cancer and 49% of those with adenocarcinoma had biomarker results available [35]. These data highlight the importance of molecular biomarkers, which have transformed the approach to lung cancer by enabling genetic characterization of the tumor and selection of targeted therapies. For example, identification of alterations such as EGFR and ALK has facilitated the use of specific treatments and may contribute to improved patient prognosis [28,36,37].

Although the proportion of patients with biomarker testing in this cohort is comparable to that reported in other contexts, only 45 patients (30.00%) received targeted therapy or immunotherapy as part of the therapeutic approach. This low coverage could be explained, among other factors, by the advanced stage of disease in some patients, which may have limited the possibility of completing molecular testing or accessing these treatments before death. Nevertheless, it could also reflect structural barriers to access to services and high-impact clinical technologies, since 29 patients (64.44%) who received these therapies did so through requests to higher institutional authorities outside the conventional protocol. This mechanism, mediated in some cases by treating oncologists or through support from nongovernmental organizations, could contribute to inequities in access to innovative treatments.

In this context, access to targeted therapies and immunotherapy should not be considered a privilege contingent upon additional administrative procedures, but rather a therapeutic option available to all individuals who meet clinical and molecular criteria. Patients with lung cancer move through a critical pathway marked by high clinical and emotional vulnerability in the context of a disease with low survival. Therefore, care protocols should systematically include molecular biomarker testing and ensure clear pathways for the availability of therapeutic alternatives aligned with each patient’s clinical and biological characteristics, in accordance with the principles of equity, timeliness, and quality that have characterized the Costa Rican health system.

Regarding diagnostic delays, a mean of 92.69 days was estimated between suspicion and biopsy-confirmed diagnosis of lung cancer. It is important to note that documented suspicion was operationalized as the earliest dated event available in the Hospital México medical record that was related to the diagnostic work-up for suspected lung cancer; therefore, this value may underestimate the actual interval, as it does not capture the previous period corresponding to symptom onset or the first general consultation. Empirical studies have described variable time intervals: in the United States, a mean time of 35.5 days (±31.6) was estimated for lung cancer diagnosis [38]; in Iran, an interval of 122 days was reported between presentation and definitive diagnosis [39]; in Spain, 82.25% of patients received the diagnosis within the first 30 days [8]; whereas in Nepal, the diagnostic interval exceeded 30 days [40].

In the analysis of diagnostic delays, it is important to highlight the relevance of the time before consultation in primary care. One study has shown that barriers such as denial, anxiety, and lack of symptom recognition may influence delays before the first consultation [41]. Therefore, strengthening the first level of care through person-centered strategies, service reorientation, and clear referral pathways is crucial. This would help build bridges between people’s life contexts, their risk factors, and the possibility of seeking care in a supported and timely manner when persistent or suspicious symptoms occur.

Regarding the interval between diagnosis and first treatment, a mean of 72.98 days (±101.73) was estimated. This is a critical aspect, as some patients ultimately receive palliative rather than curative-intent treatment, reaffirming the importance of early diagnosis and public policy interventions. According to a meta-analysis, time intervals between diagnosis and treatment in lung cancer vary according to context and patient characteristics; however, they frequently exceed recommendations from international clinical guidelines. A systematic review of waiting times reinforces the need to reduce these intervals to improve lung cancer management and prognosis [42]. In addition, delays in this phase may favor tumor growth and stage progression, which are associated with lower survival [43].

Finally, the estimated five-year survival from the first care episode related to suspected lung cancer was 1.9% (95% CI: 0.5-5.1). This estimate should be interpreted according to the selected starting point for follow-up, which was the first care episode related to suspected lung cancer rather than the date of pathological diagnosis. Therefore, it should not be interpreted as a conventional estimate of overall survival from diagnosis. The low survival observed in this cohort may reflect, at least in part, the high proportion of patients with characteristics compatible with advanced disease, diagnostic delays, nonspecific symptoms, incomplete biomarker coverage, and limited access to innovative therapies. Although these findings are consistent with the literature describing lung cancer as one of the malignancies with the lowest survival rates, the magnitude observed in this cohort highlights the need to strengthen the care pathway, particularly in contexts of advanced disease and unequal access to diagnostic and therapeutic technologies [44-48].

This study has limitations. First, its retrospective, single-center design based on medical record review may be affected by incomplete or heterogeneous documentation. In addition, physical medical records could not be accessed for 18 patients, and several variables were available only when they were explicitly recorded or ruled out in the medical record, which may have introduced information bias and variable denominators. The operational definition of documented suspicion was based on information available in the tertiary-level medical record; therefore, delays occurring before the first general consultation or before referral to Hospital México may have been underestimated. Extreme delay values should also be interpreted with caution, as they may reflect gaps in documentation, referral, or continuity of care. Survival was estimated from the first care episode related to suspected lung cancer rather than from the date of pathological diagnosis; therefore, the five-year survival estimate should not be interpreted as conventional overall survival from diagnosis. This selected time origin may also introduce lead-time-related bias and should be considered when comparing these findings with studies that estimate survival from pathological diagnosis or treatment initiation. The study did not include a multivariable survival model and was not designed to estimate independent prognostic effects. Information on performance status, such as ECOG or Karnofsky scores, was not consistently available, and AJCC stage groups were not reconstructed beyond the T, N, and M categories documented in the medical record. Similarly, the technical platforms used for biomarker testing, such as PCR, immunohistochemistry, or next-generation sequencing, were not consistently documented. Finally, this was a descriptive observational study and was not designed to establish causal associations or to adjust for all potential confounders, which limits the generalizability of the findings beyond this hospital network.

## Conclusions

A higher probability of mortality among male patients was observed in the bivariate analysis; however, this finding should be interpreted cautiously because no adjusted prognostic model was performed. This finding may reflect unmeasured biological, clinical, or timeliness-of-care differences that require further analysis. The high mortality observed may be related to the proportion of patients with characteristics compatible with advanced disease, which limits treatment options with curative intent and reduces survival. Likewise, a history of current or previous smoking remains a relevant factor in this population, underscoring the need to strengthen primary prevention, smoking cessation, and surveillance strategies for at-risk groups.

Although the use of multiple diagnostic tools was documented, including CT, radiography, bronchoscopy, and biopsy, access to molecular biomarkers continues to represent a challenge, with direct implications for the ability to select targeted therapies and immunotherapy. Delays in the care pathway constitute one of the most critical findings: the median time between suspicion and diagnosis was 34 days, but extreme values exceeding 1000 days were observed, which may reflect systemic barriers and gaps in continuity of care. The estimated five-year survival from the first care episode related to suspected lung cancer was 1.9%, highlighting the urgent need to strengthen the lung cancer care pathway, improve early detection, and ensure timely access to diagnostic testing and innovative treatments to support early clinical decisions and expand the therapeutic margin for these patients.
